# HTJoinSolver: Human immunoglobulin VDJ partitioning using approximate dynamic programming constrained by conserved motifs

**DOI:** 10.1186/s12859-015-0589-x

**Published:** 2015-05-23

**Authors:** Daniel E Russ, Kwan-Yuet Ho, Nancy S Longo

**Affiliations:** Division of Computational Bioscience, Center for Information Technology, NIH, 12 South Drive, Bethesda, MD 20892 USA; Vaccine Research Center, National Institute of Allergy and Infectious Diseases, NIH, 40 Convent Drive, Bethesda, MD 20892 USA

## Abstract

**Background:**

Partitioning the human immunoglobulin variable region into variable (V), diversity (D), and joining (J) segments is a common sequence analysis step. We introduce a novel approximate dynamic programming method that uses conserved immunoglobulin gene motifs to improve performance of aligning V-segments of rearranged immunoglobulin (Ig) genes. Our new algorithm enhances the former JOINSOLVER algorithm by processing sequences with insertions and/or deletions (indels) and improves the efficiency for large datasets provided by high throughput sequencing.

**Results:**

In our simulations, which include rearrangements with indels, the V-matching success rate improved from 61% for partial alignments of sequences with indels in the original algorithm to over 99% in the approximate algorithm. An improvement in the alignment of human VDJ rearrangements over the initial JOINSOLVER algorithm was also seen when compared to the Stanford.S22 human Ig dataset with an online VDJ partitioning software evaluation tool.

**Conclusions:**

HTJoinSolver can rapidly identify V- and J-segments with indels to high accuracy for mutated sequences when the mutation probability is around 30% and 20% respectively. The D-segment is much harder to fit even at 20% mutation probability. For all segments, the probability of correctly matching V, D, and J increases with our alignment score.

## Background

Immunoglobulins (Ig) are a family of proteins that identify and bind foreign pathogens, e.g., bacteria and viruses. Diversity in the antigen-binding region of Ig provides an appropriate immune response to the wide array of pathogens confronting individuals. This diversity is generated by VDJ recombination, which joins a Variable (V) gene segment, a Diversity (D) gene segment, and a Joining (J) gene segment from distant regions of DNA to potentially create about 10 billion different antibodies, each of which binds to a distinct epitope.

During recombination, nucleotide excision of the germline gene termini and the addition of nontemplated N nucleotides by terminal deoxynucleotidyl transferase (TdT) at the V to D and D to J junctions provide additional diversification. Furthermore, during germinal center reactions B cell receptors undergo further changes including somatic hypermutation as well as nucleotide insertions and/or deletions (indels) of various length which creates a larger potential repertoire of antibodies.

Previously the JOINSOLVER [1] algorithm was used successfully to compare an unknown VDJ rearrangement against a set of V-, D-, and J-germline sequences to provide information about gene utilization in the Ig repertoire in a wide variety of conditions including: S. aureus immune evasion [2], CDR3 characteristics and VH mutations in systemic lupus erythematosus [3], immunological memory in chronic granulomatous disease [4], rheumatoid arthritis, [5], CDR3H characterization of the fetus and neonates [6]; X-linked HyperIgM [7], and the analysis of the neutralizing HIV antibodies [8]. Unfortunately, JOINSOLVER was not designed to handle indels.

This paper addresses the challenge of both accurately aligning heavily mutated Ig rearrangements, potentially with indels, to the nearest matching V, D, and J germline gene and identifying junctional N nucleotides. We introduce a sequence alignment algorithm that approximates the results of a dynamic programming (DP) algorithm, which can save up to 98% of the computational time.

Dynamic programming algorithms have been used to align sequences since 1970 [9,10]. Typically, DP alignment algorithms align sequences by creating a matrix with the rows corresponding to the bases of one sequence, and columns corresponding to the bases of second sequence. Matrix element (*i*, *j*) is the best alignment up to the *i*th base of the first sequence and the *j*th base of the second sequence. Dynamic programming algorithms have rules that define how to initialize the matrix, how to fill matrix elements after initialization, and where to find the highest score. A traceback is kept to mark the path of the best alignment through the matrix starting from the highest score matrix element. Most DP algorithms rules allow for insertions, deletions, mismatches, and matches. A match or mutated base is a one base step forward in both sequences, corresponding to a diagonal step in the DP matrix. Insertions and deletions increment one sequence, but not the other, corresponding to a right or downward step, respectively. Typically DP algorithms have poor performance, of order O(*NM*) where *N* and *M* are the lengths of the two sequences being matched [10]. Durban [11] provides an outstanding, in depth explanation of the use of DP algorithms for sequence matching.

Previous work suggests that banding, or working along a diagonal band in the DP matrix, improves the performance of DP algorithms [12]. In the same spirit, our method uses prior biological knowledge to lock down the alignment at highly conserved motifs in V- and J-germline genes and traverses along the diagonal of the DP matrix to significantly improve the speed and accuracy of alignments. When the motifs are not found, the algorithm falls back to a more traditional DP algorithm.

The alignment of the V-segment accounts for most of the computational work. The amount of work is related to the length of the segments and the number of sequences being compared against the segment. In germline database used by JOINSOLVER, there are many more V-germline genes (289) than D- or J-germline genes (84 and 12 respectively), and the V-germline genes have the longest germline sequences (~285 nucleotides) used in the analysis. The algorithm balances the need for aligning these irregular V-segments with the need to analyze large numbers of sequences provided by next generation sequencing. A new desktop application, HTJoinSolver, is provided as an implementation of the new partitioning method.

## Methods

### Partitioning sequences using conserved motifs

**Figure 1:**
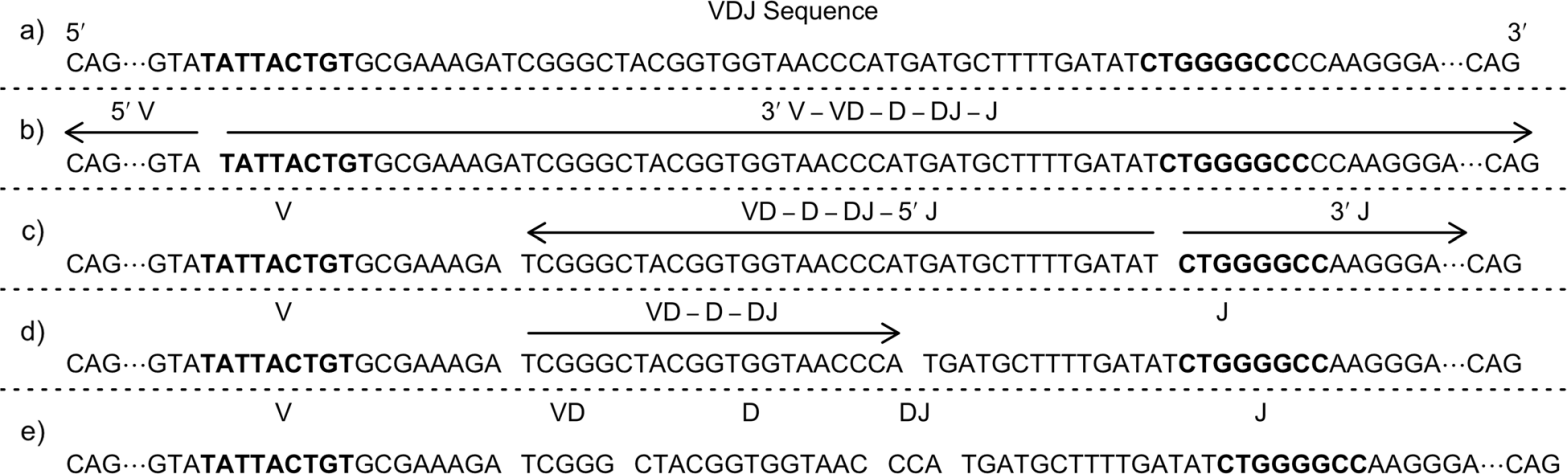
Overview of V(D)J Partitioning. Partitioning Ig VDJ rearrangements at conserved VH & JH motifs for alignment with the approximate backwards algorithm and other DP algorithms. **a)** A VDJ nucleotide sequence before subdivision and algorithm processing. The dots between CAG…GTA and GGA…CAG represent the nucleotides that are omitted for brevity. The V and J motifs, TAT TAC TGT and C TGG GG, respectively are shown in bold face type. **b)** The VDJ rearrangement is divided into 2 sections: the 5’ end of the V-segment containing codons 1–101; and the 3’ end of the V-segment, the VD junction, the D-segment, the DJ junction, and the J-segment. The 5’ end of the V-segment is aligned backwards (3’ to 5’), and the reset of the sequence is aligned forwards (5’ to 3’). The V-end is identified and the two parts of the V are merged. **c)** The rest of sequence is split just before the J motif, which is where the J-Start DP algorithm aligns the sequence to a J gene (left arrow) and determines the 5’ end of the J-segment. The V-end algorithm is also used to identify the 3’ end of the JH (right arrow). The 5’ and 3’ ends of the J are merged. **d)** A specialized local DP algorithm is used to align a D-gene within the VD-D-DJ subunit. In the figure, the V-, D-, and J-segments within each partition of the sequence are labeled. The intervening nucleotides labeled VD and DJ represent N addition nucleotides in the junctions.

Similar to the original JOINSOLVER algorithm, conserved motifs initiate the alignment process [1]. In preparation for heavy chain VDJ alignment, the rearrangements are split into smaller regions using the conserved 3’ VH-motif “TAT TAC TGT” and JH-motif “C TGG GG”. If a motif is not found, we fall back to other methods of finding the motif, which are described below. Figure 1 provides an overview of the partitioning process with an example sequence. In the figure, many of the V and J nucleotides are replaced with dots to preserve space. First the conserved motifs are found in the sequence (Figure 1a, the motifs are bold). The sequence is split just before the highly conserved 3’ V-motif (Figure 1b). The sequence on the 5’ side of the V-motif, which includes the nucleotides encoding codons 1–101 of the V-germline using IMGT numbering [13], is aligned using our approximate backwards DP algorithm (3’ to 5’). In the figure, the arrows show the alignment direction. The sequence on the 3’ side of the V-motif consists of the 3’ of the V-segment, the VD junction, D-segment, DJ junction, and the J-segment. Our V-end algorithm, an overlap DP algorithm described below, aligns the sequence on the 3’ side of the V-motif and identifies the end of the V-segment. The two parts of the V-segment are merged to produce a completely aligned V-segment. The remainder of the rearrangement consists of the unaligned VD junction, the D-segment, the DJ junction, and the J-segment.

Next the partitioning method identifies the JH segment using a highly conserved J motif in a process similar to the VH alignment (Figure 1c). The remaining unaligned sequence is split just before the J-motif. A DP algorithm is used to align this fragment to J germline genes and to identify the 5’ start of the J-segment. The V-end algorithm is used to align the 3’ end of the J-segment. The two J fragment alignments are merged to produce a fully aligned J-segment. Finally, the D-segment is matched using a specialized local DP algorithm (Figure 1d) to produce a fully partitioned and aligned rearrangement (Figure 1e). Those nucleotides that are not partitioned with the VH, D, or JH segment are considered N addition nucleotides.

### The approximate backwards algorithm

Unlike the DP algorithms commonly used for sequence alignment, the approximate backwards algorithm starts from a known point and works backwards to the start of the matrix formed by the germline gene and the unknown or query sequence. In this study, the scoring rules are +5 for a match and −4 for a mismatch; gap opening (indel formation) is penalized −30, and gap extension (continuation of indel) is penalized −1. The numbers in the matrix elements are the scores of the best alignment up to the bases corresponding to the column and row. The algorithm starts at the location of the 5’ T of the conserved V-motif in both the germline sequence and the query Ig sequence. The score is initialized to zero. The algorithm steps one base backwards, toward the 5’ end in both sequences. A traceback, which points to the previous matrix element, is kept to mark the alignment. As long as the bases match, the algorithm continues stepping in the 5’ direction.

If the bases don’t match, the algorithm considers the possibility of a mutation or an indel. The algorithm steps forward 10 bases (toward the 3’ end) and calculates a small 40 × 40 DP matrix using an algorithm similar to the Needleman-Wunsch algorithm [9,10] to find the best alignment in this small section of the sequences. The small matrix is initialized by assuming inserts, which fill the elements in last column, and deletions, which fill the bottom row. Every other element in the small matrix is the maximum of a match, a mismatch, an insertion, or a deletion using the scoring rules. The tracebacks maintain the alignment through the small matrix. By calculating all elements of the small matrix, the algorithm determines the best alignment through the matrix taking insertions, deletions, mutations, and matches into account. The algorithm continues aligning from the highest scoring entry in first row or column of the matrix.

**Figure 2:**
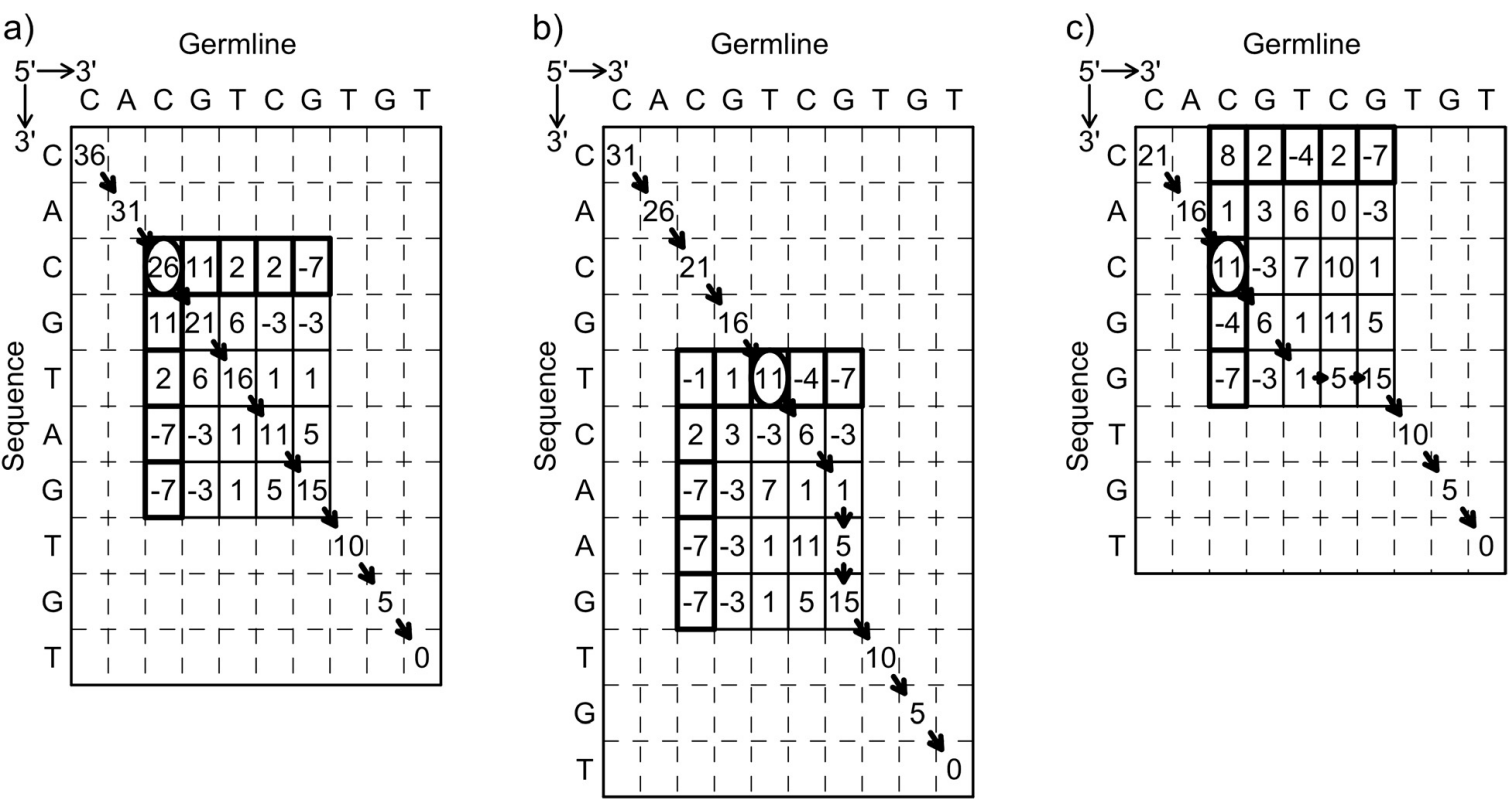
Matrix calculation of the alignment score for a sequence with a mutation or indel. Matrix calculation of the alignment score for a sequence with a mutation or indel. **(a)** Matrix for a single nucleotide mismatch. **(b)** Matrix with a two-base insertion (CG > CAAG). **(c)** Matrix with a two-base deletion (TC > −−). The dynamic programming matrix for the approximate backwards algorithm begins at the initial T of the VH-motif (last row and column, score = 0). The algorithm goes backwards along the diagonal until it hits a mismatch, in which the algorithm backs up a step and generates a submatrix (solid lines). The algorithm can choose to step up (deletion), step to the left (insertion), or continue diagonally (match/mismatch). For a deletion or insertion, the score initially decrease by 10, but subsequent indels have a score decrease of 4. Matches increase the score by 5, and mismatches decrease the score by 4. The maximum score in the first column or row (bold box) is selected (circled). The algorithm continues stepping backwards on the diagonal. Backtraces are shown as arrows, and label the alignment of the sequences.

Figure 2 demonstrates how the algorithm aligns sequences. In the examples provided in Figure 2, the starting point uses the most 5’ T from the conserved V-motif, TAT TAC TGT. The germline and unknown sequences match for the next 3 bases (G, T, and G), so the algorithm walks along the diagonal in the 5’ direction of the DP matrix. When a mismatch occurs, it traces back and calculates the score over a small rectangular submatrix. In Figure 2a, which corresponds to a single C to A mutation, the high score traces straight along the diagonal. In Figure 2b, which corresponds to a two base insert (AA), the traceback has two steps down. Figure 2c, which has a two base deletion (TC), the traceback has two right steps. In all the examples in the figure, arrows represent the trace backs, the top rows and first columns are shown in bold, and the highest scores are circled. The algorithm continues stepping back in the 5’ direction along the diagonal from the high scoring element. Finally, the algorithm terminates at the top left matrix element. To conserve space in the figure, the algorithm traced back 1 step and a 5 × 5 square matrix was calculated. However, these parameters are too small to avoid falling into local maxima, thus, in actuality, the algorithm uses a 40 × 40 square matrix on a mismatch, and traces back 10 steps.

After completing the alignment, sequences with indels are re-aligned from 5’ end of the alignment to the 3’ end of the V-motif. The two alignments are compared for consistency. This quality control step catches suboptimal alignments caused by local maxima.

### 3’ V-end alignment algorithm

After aligning the 5’ end of the V-segment, we align the 3’ of the V-segment starting at the conserved VH-motif. The V-end alignment algorithm, which is a type of overlap DP algorithm [11], begins at the first “T” in the VH-motif for both the query and germline sequences. A full matrix calculation is required, however the matrix is fairly small because only a few nucleotides occur after the V-motif in the V-germline genes. The 3’ end of the V-segment is defined by the maximum score, which can occur anywhere in the matrix. The 5’ J-end alignment uses the same algorithm, starting at the “C” from the conserved JH-motif.

### J-start alignment algorithm

The start of the J-segment is aligned using a DP algorithm similar to a Needleman-Wunsch algorithm; however, the maximum score can be anywhere in the DP matrix, and the algorithm is run backwards from the J-motif. The maximum score corresponds to the start of the J-segment. The alignment is determined by tracing back from the highest scoring matrix entry.

### D matching alignment algorithm

The D alignment uses a local alignment algorithm similar to a Smith-Waterman algorithm. A match is given a score of +1, however a mismatch sets the score back to 0. When the score = 3, the algorithm looks back 4 steps to check for a potential mutation, if the score before the match start is greater than 3, the current score is increased by the previous score. This allows a mismatch to be recognized as a mutation in the D-segment if and only if there are at least 3 consecutively matching bases on both sides of the mutation. The probability of randomly matching 3 bases is around 1.5% or 3% on either side of the D-segment, and 3 was chose to keep the probability of random matches below 5%. The termination condition is the maximum score anywhere in the matrix. These modifications to the Smith-Waterman algorithm maintain the basis of the original JOINSOLVER, matching D-segments based on consecutive matching bases.

### Falling back to other algorithms

In the event, the V-motif is not found in the sequence, the algorithm looks for the motif with one mutation. If the motif with a single mutation is found, the approximate backwards algorithm is run using the mutated motif location. Because the J-motif is shorter, mutations are not allowed in the J motif.

When the location of the V-motif is wrong, the alignment is very poor resulting in a low score. Setting a score threshold of 3.1/base effectively catches an incorrect motif location. Before relinquishing to a slower algorithm, one last attempt is made to identify the location of the motif. The sequence is aligned to the first germline sequence in our database (IGHV1-18*01). The motif location from this alignment is used to align the query sequence to all other germline genes to determine the best match or alignment. If the highest scoring alignment is still below 3.1/base, we run an overlapping sequence DP algorithm for the V or a Smith-Waterman algorithm for the J alignments. Our only modification is that the end of the V- or J-segment occurs at the maximum score anywhere in the matrix, not just in the last row or column.

### Simulations

Artificial VDJ rearrangements were generated by randomly recombining a VH-, D-, and JH-germline gene from the JOINSOLVER germline database. A random number of terminal nucleotides from the 3’ V, 5’ & 3’ D and 5’ J were removed to mimic exonuclease activity. Various random numbers of nucleotides were added to the V-D and D-J junctions to mimic TdT activity. In our simulation, each base had a fixed probability of being mutated (referred to as the mutation probability). The mutation probability should not be confused with the mutation frequency, which is the number of mutations is a sequence divided by the sequence length. The number of indels in our simulation was randomly selected using a distribution that heavily favors one indel per sequence, *P(n) = cne*^*-3(n-1)*^, where *n* is the number of indels, and *c = (1-e*^*3*^*)*^*2*^ is a normalization constant. In our simulator, the indel length is selected from a Poisson distribution. If a length of zero is selected, the distribution is re-sampled. Table 1 shows the default parameters of our simulations. Our simulations do not intentionally mimic the molecular mechanism of Activation-Induced Cytidine Deaminase (AID), which specifically targets the G in RGYW motifs and the C in WRCY motifs or the AT bias introduced by mismatch repair mechanisms [14].

**Table 1:** **Default parameter used in simulations, unless otherwise mentioned**

**Excision**							**Junction**			**Mutation**	
	V		D		J			μ	σ	Probability	(0-95%)
	μ	σ	μ	σ	μ	σ	VD	2	1	Indel	
Start (5’)	8	2	5	1	7	2	DJ	2	1	Number	Random (usually 1)
End (3’)	2	1	2	1	13	3				Mean Length	4

The amount of excision and addition for an artificial rearrangement are randomly selected from a normal distribution. The μ and σ are the means and standard deviations. Indel lengths are selected from Poisson distribution, therefore only a mean is needed. The number of indels is selected from a distribution of the form *P*(n) = cne^-3n^ to require an indel, but heavily weight n = 1. Location of indels are randomly selected.

Two sets of simulations were performed. In the first simulation, 10,000 rearrangements were generated with mutation probabilities ranging from 0% to 95% in increments of 5% throughout the VDJ sequence. Using these rearrangements, the approximate backwards algorithm was compared against a complete DP algorithm, to calculate the success rate. The second set of simulations with 1,000 rearrangements engineered with a mutation probability of 3.5% was performed to compare HTJoinSolver with the original JOINSOLVER using the original JOINSOLVER germline database. The results of an alignment are considered a successful match to a germline if the algorithm returns the correct gene; the exact allele is not required.

### Comparison with a standard dataset of biological VDJ rearrangements

Additionally 13,153 human sequences from the Stanford.S22 dataset [15] were analyzed to compare HTJoinSolver with several other frequently employed algorithms. Briefly, the Stanford_S22 data were produced by 454 sequencing of peripheral blood mononuclear cells from a single donor. The S22 individual genotype was determined using an individual analysis of iHMMune-align [16] results. The Stanford_S22 dataset and the S22 genotype form a standard dataset to evaluate the performance of VDJ partitioning algorithms. An online evaluation tool, Evaluation of IGH partitioning tools ([17], http://www.emi.unsw.edu.au/~ihmmune/IGHUtilityEval/evalForm.html) compares the performance of VDJ partitioning tools on the Stanford_S22 dataset. As a performance metric, the tool reports the percentage of V, D, or J assignments to germlines that are not present in the predetermined S22 genotype.

### Germline database

The germline genes used in HTJoinSolver are IMGT reference sequences [18,19], www.imgt.org/download/GENE-DB/IMGTGENEDB-ReferenceSequences.fasta-nt-WithGaps-F+ORF+inframeP, downloaded July 30, 2014). HTJoinSolver provides methods to download and reformat germline genes from IMGT. These methods allow the user to maintain an up-to-date library of germline genes usable by HTJoinSolver. When comparing HTJoinSolver with JOINSOLVER, we used the JOINSOLVER germline database, which was derived from IMGT and included pseudogenes.

### Access to HTJoinSolver

HTJoinSolver is available for public use, and can be downloaded from https://dcb.cit.nih.gov/HTJoinSolver.

## Results

**Figure 3:**
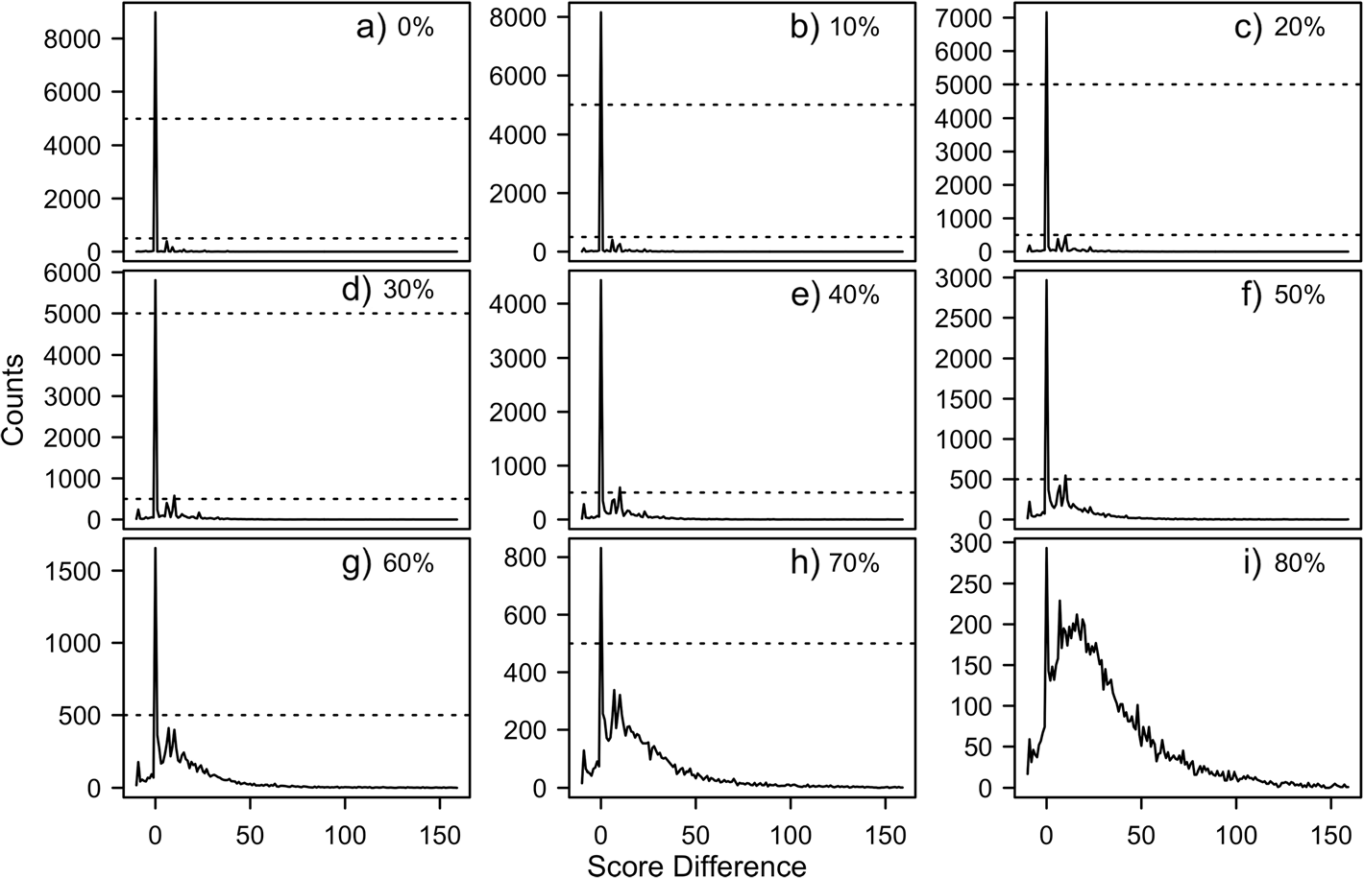
The difference in scores between the approximate backwards algorithm and an overlapping DP algorithm. The difference in scores between the approximate backwards algorithm and an overlapping DP algorithm for mutation probabilities between 0 and 80%. The counts on the y-axis are the number artificial rearrangements and the score difference is on the x-axis. In **(a-d)**, the sharp peak at zero shows that the difference in score between the two algorithms is zero for most of the sequences. As the mutation probability increases **(e-i)**, the scale for the counts changes as the difference between the algorithms becomes more apparent. The dotted lines indicate a constant count level at 5000 and 500 across the figure. Negative score differences indicate the approximation had a higher score than the full algorithm.

The approximate backward algorithm was designed to quickly estimate the alignment score of a complete DP algorithm for overlapping sequences without sacrificing accuracy. Figure 3 shows the score differences between the approximate algorithm and the full DP algorithm for simulated sequence alignments with mutation probabilities ranging from 0% to 80%. Score differences between the approximate backwards algorithm and the full DP algorithm for sequences with no mutations (Figure 3a) were very rare. For mutation probabilities up to 30% (Figure 3b-d), which far exceeds the ~6% mutation frequency of average memory B cells [7] and includes the elevated nucleotide mutation frequency of some HIV antibodies [20], a sharp peak at zero indicates that the approximate algorithm replicates the expected score very well. As the mutation probability increases (Figure 3e-i), slight differences in scores between the two methods cause the distribution to spread. The dashed lines are presented to emphasize the differences in the Y-axis caused by the wider distribution. These results show that the approximate backwards algorithm can provide a good estimate of the alignment score calculated by a full DP algorithm.

The results of the HTJoinSolver approximate algorithm were then compared to the original JOINSOLVER V-alignment algorithm. Although the JOINSOLVER algorithm was not designed to identify indels, large portions of the rearrangement can align correctly up to the position of the indel, but the remaining V-segment was mismatched. Unfortunately, the results were not usable for mutation analysis because the offset alignment appeared as a region of high mutation instead of a single indel. Adding V-segment indels to our simulations hurt the overall performance of the original JOINSOLVER. With a 3.5% mutation probability and simulated indels, the original algorithm selected the correct V-germline 61% of the time, whereas the new algorithm selected the correct V-germline over 99% of the time. The percent of success for the old vs. new algorithms were 73% vs. 91% and 98% vs. 99% for the D- and J-germlines, respectively. These simulations show that the approximate backwards algorithm performs as well or better than the original JOINSOLVER algorithm.

**Figure 4:**
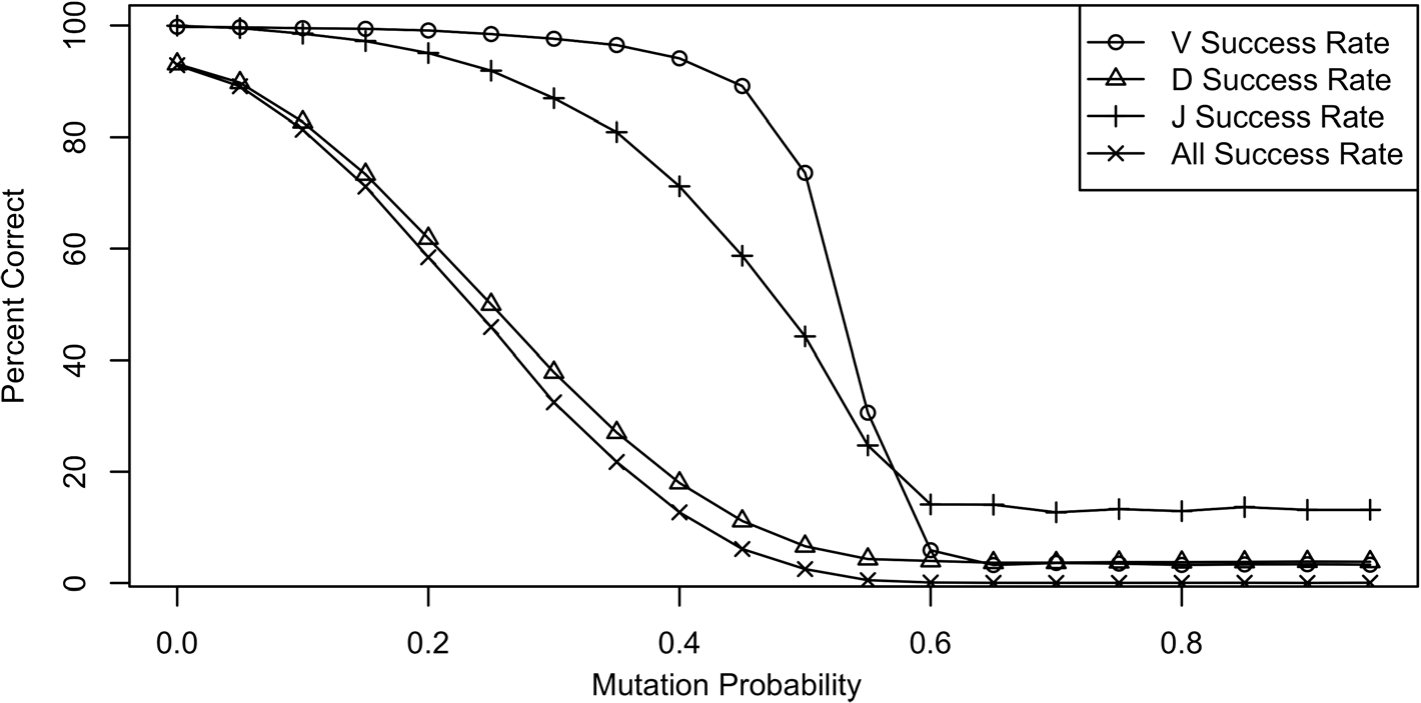
The success rate for simulated sequences as a function of the mutation probability. At each mutation probability, 10,000 artificial rearrangements were generated. The success rate is the percentage of rearrangements with correctly identified V- (circles), D- (triangles), and J- (pluses) germlines. The All Success (X) line is the percent of rearrangements with all gene utilization correctly identified.

As the mutation probability increases, sequence alignment becomes more difficult. The effect of the mutation probability on the success rate can be seen in Figure 4. The success rates for the V, D, and J have sigmoidal curves. The V-segment has a success rate of around 95% even when the mutation probability is approximately 40%. The sigmoidal curve decreases sharply from around 95% success rate to below 10% as the mutation probability changes from 40% to 60%. A leveling off of the success rate at around 3% is due to random matching to the correct V-germline. The sigmoidal shape of the J has a gentler slope and levels off at around 13%, which is less than the 17% expected from randomly matching J-germlines. However, if the highly mutated sequences matched the wrong V, it is possible that there are no nucleotides left for a J match. No J germline would be assigned, which would tend to decrease the J success rate.

 The D-segment, which is relatively short, is the most difficult segment to identify and align. The D-segment nucleotides must be found within the CDR3, which is the most complex region of the VDJ rearrangement. In order to locate the CDR3 5’ and 3’ boundaries the algorithm must first correctly identify the 3’ end of the V-segment and the 5’ end of the J-segment whose nucleotide sequences may differ from the germline gene if nucleotide excision and/or mutation has occurred. N addition may be present or absent and varies in the number of nucleotides flanking the D-segment. Furthermore, D-segment mutations decrease the number of consecutively matching D-nucleotides. Thus, the D alignment success rate drops off almost immediately as the mutation probability increases. The curve in Figure 4 labeled “D Success Rate” levels off at around 4%, which is around what is expected by randomly matching D-germlines. The success rate drops from 93% at a mutation probability of zero to 89% at a mutation probability of 5%. The solid line with ‘X’ marks labeled “All Success Rate” corresponds to matching the full VDJ rearrangement. This curve strongly resembles the D success curve, reinforcing the fact that the D alignment is the most difficult and performance-limiting step.

**Figure 5:**
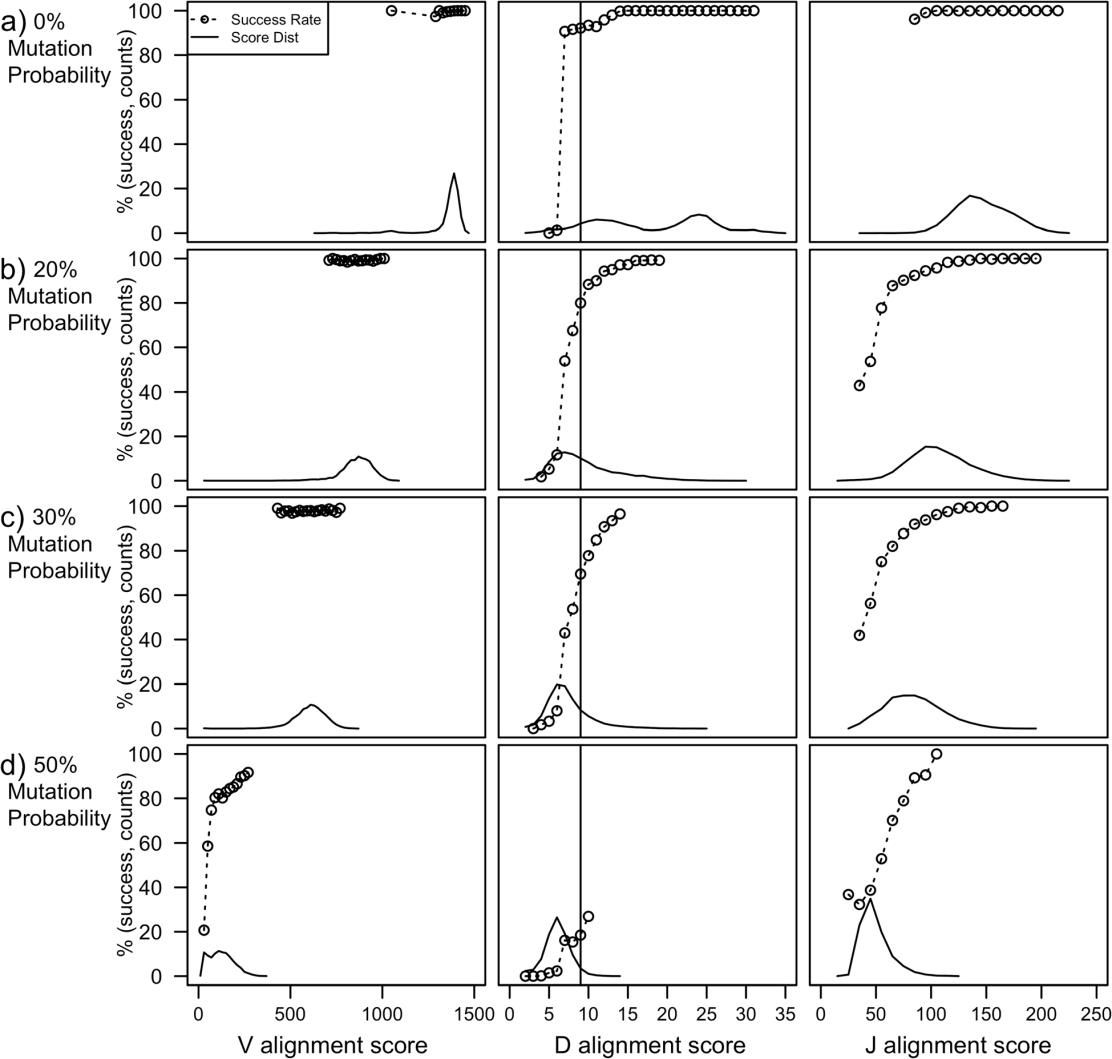
The success rate and score distribution of V, D, and J alignments for simulated rearrangements with mutation probability: **a)** 0% **b)** 20% **c)** 30% and **d)** 50%. The success rate is a function of the alignment score and mutation probability. The success rate (circles with dashed lines) is the frequency at which a V-, D-, or J-alignments in the simulated sequences are correct. The score distribution is calculated from the number of times an alignment score occurs in 10,000 simulated sequences. Scores that occur less than 10 times (out of the 10,000 simulations) are not shown to prevent discontinuities. V-segments scores are normalized by the length to account for differences in the size of the V-germlines, which may have 5’ or 3’ truncations. The solid vertical line in the D-alignment score at score = 9 corresponds to the suggested minimum length for the size of a D-match in JOINSOLVER. The increase in the success rate of low scoring J-matches is a result of ties in which most or all J germlines are selected.

In our simulations, the alignment scores tend to decrease as the mutation probabilities increase. However, for most biologically relevant situations, the success rate remains high. Figure 5 shows the success rate for V, D, and J matching of simulated sequences with mutation probabilities of (a) 0%, (b) 20%, (c) 30%, and (d) 50% as a function of score. This simulation is important, because in real sequences, the mutation frequency is unknown. The success rate for V-, D- and J-rearrangement alignments improves as the score increases and is inversely related to the mutation probability. The score distributions are presented to allow the reader to focus attention to regions where the success rate is most relevant. As seen in the figure, as the mutation probability increases, the alignment score distribution shifts to smaller scores for the V, D, and J alignments. However, the success rate remains high at the peak of the score distribution (i.e. where most of the counts are, the success rate is high). The success rate is only shown for scoring bins that have more than 100 counts in the score distribution. The first column in the figure is the success rate for V-matching. For V-alignments, the scores decrease from approximately 1500 to 700 going from a mutation probability of 0% to 20%, however the success rate remains near 100%.

The second column in Figure 5 is the success rate for the D-segment. The solid vertical line is at alignment score equals nine, marking the shortest acceptable D-score suggested in the original JOINSOLVER paper [1]. The effect of mutation is pronounced. As the mutation probability increases, fewer sequences have scores that cross the 9-base threshold needed to avoid random matches. When the mutation probability gets high, even though random matches of length 9 are rare, they occur as likely as real matches. Therefore, the success rate drops even at scores as high as 9. The J-segment success rates are shown in the right column of Figure 5. For low mutation probabilities, the J-segment is well identified. As the mutation probability increases to 20%, the success rate drops significantly for low scoring alignments.

The “Evaluation of IGH partitioning using inferred genotypes tool” [17] was used to compare the performance of HTJoinSolver with other applications. The results are shown in Table 2. The initial evaluation was performed using the original JOINSOLVER germline database. The comparison of the D-segment alignment was problematic since HTJoinSolver provides D results only when the score is greater than 9 for the D length. Many of the errors were caused when short D-segments rejected by HTJoinSolver. To overcome the restriction in D length, the S22 dataset was repartitioned using HTJoinSolver so that D results were always provided, even when the score is less than 9. The rows in Table 2 are labeled (D ≥ 9) for our results that do not include score less than 9, and (All D) for the all the D results regardless of the score. The D score does not affect the V and J assignment. For both cases the results compare favorably to those reported in [17] using 7 different alignment applications. After the evaluation, the germline database was updated to use the IMGT reference sequences. The re-evaluated results are found in the rows labeled (Updated). By switching the germline database, the performance of the algorithm decreased according to the evaluation tool. If the performance tool is evaluating the algorithm, switching the germline database should not effect the evaluation.

**Table 2:** **Evaluation of IGH partitioning using inferred genotypes tool**

**Database**		**V (%)**	**D (%)**	**J (%)**	**Total (%)**
Original	D ≥ 9	3.60 (0.74)	2.30 (2.14)	0.42 (0.0)	6.17
	All D	3.60 (0.74)	1.11 (0.99)	0.42 (0.0)	4.91
Updated	D ≥ 9	6.80 (0.28)	0.71 (0.59)	0.53 (0.0)	7.88
	All D	6.80 (0.28)	1.25 (1.07)	0.53 (0.0)	8.43

HTJoinSolver alignment of the S22 dataset of human VDJ rearrangements. The percentage of selected germline alleles that is not present in the S22 genotype. In parentheses is the percentage of genes, not just allelic variants, not present in the genotype. HTJoinSolver was evaluated using the original JoinSolver database and a more recently updated germline database from IMGT. The total column is the percentage of V-, D-, or J-germline allele that is not present in the genotype.

## Discussion

High-throughput sequencing of B-cell receptor VDJ rearrangements produces vast numbers of sequences and requires extremely fast algorithms for VDJ alignment. The original JOINSOLVER algorithm was fast and accurate, but was not designed to handle indels. One of the main reasons for creating HTJoinSolver was to handle alignments with indels, while maintaining or improving the speed. Aligning a sequence to approximately 300 VH-germlines takes about 1.7 second on a 2.93 GHz Intel Core i7 iMac. Our approximate algorithm takes around 207 ms. Falling back to standard methods can take over 7x longer. One million lightly mutated sequences (simulation parameters in Table 1) took approximately 6 hours to run on 40 threads on a 4 × 12 core Opteron 6172 2.1 GHz computer.

The increase in speed occurs because we use the available biological information to avoid unnecessary calculations. We know that the highly conserved motif “TAT TAC TGT” begins at codon 102, and we know that insertions or deletions occur in less than 5% of the expressed repertoire, and are usually less than 10 bases [21]. Given these pieces of information, the algorithm calculates only a small fraction of the total DP matrix, even at high mutation probabilities. Our approximate solution was successful at replicating the score of a complete DP matrix.

When the motif is not found, the algorithm makes several attempts to align the sequence before falling back to the complete overlapping DP algorithm. However, without knowing the position of the motif, we lose the increased efficiency that comes with the use of this prior information.

A potential problem can occur if a sequence has a long stretch of mismatches that is longer than the rectangular block matrix used to determine if a mismatch is a mutation or an indel. The algorithm may incorrectly fall into a local maximum, (i.e. not seeing enough of the sequence to find the actual best alignment) and may be incorrectly aligned. Usually, the score threshold will catch the incorrect alignment, and fall back to the overlap DP algorithm. If it is known beforehand that long stretches of indels or mutation will occur, increasing the block size may improve the performance. The larger the block size, the less likely the algorithm will fall into a local maximum requiring a fall back algorithm. However, the price for a large block size is increased computation time.

We were not surprised by the comparison between the original JOINSOLVER and HTJoinSolver. We wanted to show that the HTJoinSolver can perform complete alignments of VDJ rearrangements containing indels. Therefore, all of our simulations included indels, which hindered JOINSOLVER’s performance. The increased success rate of the improved algorithm is due to the ability to identify the size and position of indels. The D and J results were very similar because the DP algorithms used to align the D and J regions resemble the original JOINSOLVER algorithms, which were designed to prevent random D matching at the 95% confidence level [1].

The ability to successfully match a sequence to a germline is heavily affected by the length of the V-, D-, or J-segment and the mutation probability. Matching V-segments is easiest because longer germline sequences are available for comparison. As the mutation probability increases, the success rate drops. Both of these cases can be explained by the loss of information from the original germlines by truncation and mutation. Additional studies on whether the success rate can be explained by a noisy-channel model could give additional insights into the theoretical performance of any alignment algorithm.

Germline sequence similarity presents an additional challenge for HTJoinSolver, especially for aligning heavily processed sequences. The problem is compounded by the fact that the D-germline sequences can be similar to the 5’ end of the J-germline sequences. A small fraction of our simulations show a VH-JH distance of zero, even though we know a D-germline is present along with VD and DJ junctions. This ambiguity cannot be resolved without additional information.

When compared against the original JOINSOLVER algorithm using the Evaluation of IGH partitioning using inferred genotypes tool, we see an improvement in the V, D, J, and overall results. However, the improvement is small because the sequences are minimally mutated. The median mutation frequency for the S22 dataset is 0% and the mean is 0.9%.

The results of the evaluation tool are highly dependent on the set of germlines used in the comparison. When compared against other partitioning tools using the original JOINSOLVER database, the results show that HTJoinSolver makes fewer V, D, and J assignment to germlines and alleles not present in the S22 genotype than the tools in the study by [17]. When the germline database was updated, the resulting evaluation changed. More V-alleles not in the S22 genotype where matched, hurting V and total performance.

The development of an evaluation tool with a standard dataset is an important first step, but without a better method of assessing the quality of results, and a standard library of germlines, comparison between partitioning tools remains difficult. We have shown that for the original JOINSOLVER® database, always including the highest scoring D alignment, even when we would not recommend using the alignment, improves the partitioning performance according to the evaluation tool. Furthermore, providing a D-segment that occurs in the S22 genome will always improve the performance as measured by the evaluation tool, even if the D-segment is very likely to be wrong. Relaxing the criteria for including a match (e.g., allowing low quality D-matches) should not have improved results of the evaluation. After updating the germline database, the performance for the V-segment dropped. With more allelic variants to choose from, the system is more likely to match previously unknown allelic variants causing the evaluated performance to decrease. Improvements in the D alignment can be explained by the removal of the reverse germlines from the database when we switched from the JOINSOLVER germline database to the IMGT reference database for HTJoinSolver. Interestingly, always including the highest scoring D-germline, regardless of whether the score is high enough, does not improve the D-segment performance using the updated database. Evaluation results for the same algorithm changed because of differences in the germline database and not on algorithmic differences.

These results suggest that evaluating a partitioning tool based solely on whether or not a germline occurs in an assumed genotype is not sufficient. Consider a partitioning algorithm that only assigned IGHV1-3*01 to all query sequences. Since IGHV1-3*01 is in the S22 genome, 0% of the sequences would come from outside the S22 genome. The partitioning algorithm would be evaluated as perfect. However, this algorithm would not make a useful tool. Whereas the evaluation tool is a good idea, currently the tool is too sensitive to similarity between the S22 genome and the germlines present in the partitioning tool’s database.

## Conclusions

All of the simulations and the comparison with the partitioning tool evaluation software show that HTJoinSolver can identify V-, D-, and J-segment to very high accuracy for reasonably mutated sequences. As the mutation probability increases, alignment becomes more difficult but problematic only for unnaturally highly mutated or truncated sequences. The V-segment is identified very accurately for most biologically relevant cases. The J-segment is fairly well identified from 0-20% mutation probability but above 20% the success rate falls below 90%. At the 20% mutation probability, the D-segment has about a 55% chance of being correct. Regardless of the partitioning software, biologists should be skeptical of short D-matches to highly mutated sequences, even if mutations are not seen in the D.
